# Single session of high-intensity focused ultrasound for localized prostate cancer: treatment outcomes and potential effect as a primary therapy

**DOI:** 10.1007/s00345-013-1215-z

**Published:** 2013-11-23

**Authors:** Kazumasa Komura, Teruo Inamoto, Tomoaki Takai, Taizo Uchimoto, Kenkichi Saito, Naoki Tanda, Junko Kono, Koichiro Minami, Hirohumi Uehara, Yutaka Fujisue, Kiyoshi Takahara, Hajime Hirano, Hayahito Nomi, Toshikazu Watsuji, Satoshi Kiyama, Haruhito Azuma

**Affiliations:** 1 Department of Urology, Osaka Medical College, 2-7 Daigaku-machi, Takatsuki City, Osaka 569-8686 Japan; 2 Department of Urology, Hirakata City Hospital, Hirakata City, Osaka Japan

**Keywords:** HIFU, Localized prostate cancer, Single-session treatment, Outcome

## Abstract

**Purpose:**

To investigate the treatment outcomes of a single-session high-intensity focused ultrasound (HIFU) using the Sonablate^®^ for patients with localized prostate cancer.

**Methods:**

Biochemical failure was defined according to the Stuttgart definition [a rise of 1.2 ng/ml or more above the nadir prostate-specific antigen (PSA)] and the Phoenix definition (a rise of 2 ng/ml or more above the nadir PSA). Disease-free survival rate was defined using the Phoenix criteria and positive follow-up biopsy.

**Results:**

A total of 171 patients were identified. Fifty-two (30.4 %) patients were identified to be with D’Amico low risk, 47 (27.5 %) with intermediate risk, and 72 (42.1 %) with high risk. In the median follow-up time of 43 months, there was 44 (25.7 %) and 36 (21.1 %) patients experienced biochemical failure for Stuttgart and Phoenix definition with mean (±SD) time to failure of 17.8 ± 2.1 and 19.4 ± 2.3 months, respectively. A total of 44 (25.7 %) patients were diagnosed as disease failure. Cox multivariate analysis revealed PSA nadir level (PSA cutoff = 0.2 ng/ml; HR = 9.472, 95 % CI 4.527–19.820, *p* < 0.001) and D’amico risk groups [HR = 3.132 (95 % CI 1.251–6.389), *p* = 0.033] were the predictor for failure in single-session HIFU.

**Conclusions:**

Single-session HIFU treatment using the Sonablate^®^ seems to be potentially curative approach. When treated carefully with neoadjuvant hormonal therapy or preoperative transurethral resection of the prostate, higher-risk disease might be able to choose this minimally invasive procedure as primary therapy.

## Introduction

Prostate cancer is the most common cancer and leading cause of cancer death in men. Now varying treatment options are available for patients with localized prostate cancer. Radiotherapy (RT) with external beam radiation (EBRT) or brachytherapy is more widely used in the treatment of men aged over 65 years and seems to be the most famous less invasive therapy [1]. High-intensity focused ultrasound (HIFU), which is a non-surgical, minimally invasive treatment option using ablative technology, was developed in the 1990s and is now becoming an alternative to radiation therapy [2]. To date, durable cancer control outcomes of HIFU treatment have been reported with high volume cohort [3, 4], which were comparable to the other modalities such as EBRT [5]. Blana et al. investigated the biochemical events that best predicted clinical failure for patients treated with HIFU which derived Stuttgart definition; reaching a threshold of prostate-specific antigen (PSA) nadir level +1.2 ng/ml [6]. This definition is now becoming a widespread biochemical indicator for patients after HIFU in the recent studies, even though being more strict criteria compared with Phoenix definition; PSA nadir level +2.0 ng/ml for patients treated with EBRT [7]. Nevertheless, the role of HIFU as primary therapy for patients with prostate cancer is still controversial. In particular, repeated administrations of HIFU after positive follow-up biopsy, which is explained as new sessions of HIFU, potentially make the assessment of treatment efficacy difficult. Data reported so far a mixing result of single session and multiple sessions. Thus, it is clinically meaningful to evaluate the single-session HIFU treatment outcome. Recently, several reports that assessed treatment outcome for the single-session HIFU using the Ablatherm^®^ have been reported [8, 9]; however, there is no available reports using the Sonablate^®^ device. In this report, we first focused on the treatment outcome of single-session HIFU using the Sonablate^®^ and clarified predictor for treatment failure after single-session HIFU treatment.

## Materials and methods

### Equipment

All patients were treated with the Sonablate^®^ HIFU device (Focus Surgery, Indianapolis, IN, USA). The transrectal HIFU probe uses double transducer technology with low-energy ultrasound (4 MHz) for real-time imaging of the prostate and delivery of high-energy ablative pulses (site intensity 1,300–2,200 W/cm^2^).

### Patients

The inclusion criteria for treatment in our institution were as follows: clinical stage T1-T2N0M0 biopsy-proven localized prostate cancer, prostate volume at diagnosis ≤50 ml, and no previous treatment for prostate cancer with curative intent. All patients were followed at least 24 months. This study was approved by the local institutional review board. Between 2004 and 2008, 180 consecutive patients undergoing HIFU at our institution were enrolled into a database. We analyzed the data of 171 patients who underwent single-session HIFU as the primary therapy with curative intent, excluding the data of 9 patients who were treated for salvage.

### Pre-HIFU treatment protocol

Patients were offered neoadjuvant hormonal ablation (NHA) to reduce the prostatic volume and facilitate delivery of high-energy ablative pulses throughout the prostate when the initial size of the prostate was >35 ml. Any hormonal therapy was discontinued at the time of the HIFU. The prostatic volume was evaluated again immediately before HIFU. Transurethral resection of the prostate (TURP) was performed before HIFU to resect calcifications within the prostate, which would disable ablative pulses from reaching the targeted focus.

### Follow-up

The follow-up examinations included digital rectal examinations (DRE), and PSA measurement every month during the first 6 months after treatment and every 3 months thereafter. A follow-up control octant biopsy was recommended to all patients 3–6 months after the treatment, and was also performed forcibly to all patients not achieved PSA level of 1.0 ng/ml at 6 months after the HIFU. Biochemical failure was defined according to the Stuttgart definition (a rise of 1.2 ng/ml or more above the nadir PSA) [6], which was generated for patients treated only with HIFU, and the Phoenix definition (a rise of 2 ng/ml or more above the nadir PSA) [7], derived from the experience with EBRT. Disease-free survival rate (DFSR) was evaluated using the definition for disease failure, which was defined according to the Phoenix criteria: a rise of 2 ng/ml or more above the nadir PSA (biochemical failure), positive follow-up biopsy, or the administration of salvage treatment including second session of HIFU. In the present study, no patient received adjuvant hormonal therapy or any other salvage therapy including second-session HIFU treatment before the diagnosis of biochemical failure and positive follow-up biopsy. Therefore, disease failure was simply defined as PSA nadir +2 ng/ml or positive follow-up biopsy. We applied the two risk classification (i.e., D’Amico risk groups [10] and National Comprehensive Cancer Network (NCCN) risk groups [11]) to compare the treatment outcome with previous studies.

### Statistical analysis

Continuous parametric variables were reported as the mean value ± standard deviation (SD). Continuous nonparametric variables were presented as the median value and interquartile range (IQR). The unpaired t test and the Mann–Whitney *U* test were used for quantitative parametric and nonparametric variables, respectively. Chi-square tests were conducted to assess the differences of the distributions between the clinicopathological parameters. The log-rank test was used to compare the curves based on Kaplan–Meier models. A multivariate Cox proportional hazards regression model was used to estimate the prognostic relevance of clinicopathological variables. Associations were regarded as significant if *p* < 0.05, and all *p* values were two-sided. All data were analyzed with the use of the Statistical Package for the Social Sciences software, version 12.0 (SPSS Inc, Chicago, IL).

## Results

### Patients’ characteristics

Table 1 summarizes the clinical and pathologic characteristics of 171 patients included in the analysis.

**Table 1:** Patient population (*n* = 171)

Median follow-up [mo] (IQR)	43 (30–55)
Mean + SD age	68.3 ± 7.0
Median PSA [ng/ml] (IQR)	7.7 (5.8–12.6)
Mean + SD prostatic volume [ml]	20.1 ± 7.6
Clinical stage (%)	
cT1c	47 (27.5)
cT2a	51 (29.8)
cT2b	40 (23.4)
cT2c	33 (19.3)
Gleason score (%)	
5 or less	9 (5.3)
6	83 (48.5)
7	37 (21.6)
Greater than 7	42 (24.6)
D’amico risk groups (%)	
Low risk	52 (30.4)
Intermediate risk	47 (27.5)
High risk	72 (42.1)
NCCN risk groups (%)	
Low risk	52 (30.4)
Intermediate risk	66 (38.6)
High risk	53 (31.0)
NHA (%)	
No	95 (55.6)
Yes	76 (44.4)
Median duration of NHA [month] (IQR)	3 (3–5.75)
TUR before HIFU (%)	
No	115 (67.3)
Yes	56 (32.7)

*SD* standard deviation, *PSA* prostate-specific antigen, *IQR* interquartile ranges, *NCCN* National Comprehensive Cancer Network, *NHA* neoadjuvant hormonal ablation, *HIFU* high-intensity focussed ultrasound, *TUR* transurethral resection

### Survival rates

The overall and cancer-specific survival rates at 5 years were 98.8 and 100 %. The metastasis-free survival rate at 5 years was 99.4 %.

### Biochemical and disease-free survival

Table 2 summarizes biochemical-free survival rates at 3 and 5 year. Stuttgart definition and Phoenix definition are utilized. Patients were stratified according to risk groups including D’Amico and NCCN. There was 44 (25.7 %) and 36 (21.1 %) patients experienced biochemical failure for Stuttgart and Phoenix definition with mean (±SD) time to failure of 17.8 ± 2.1 and 19.4 ± 2.3 months, respectively.

**Table 2:** BFS and DFS probability in 171 patients after HIFU according to risk groups

	Mean ± SE BFS probability						Mean ± SE DFS probability		
	Stuttgart definition			Phoenix definition					
Variables	3 years	5 years	*p* value	3 years	5 years	*p* value	3 years	5 years	*p* value
No. of patients at risk	63	12		68	11		67	11	
All cohort	0.72 ± 0.04	0.62 ± 0.05		0.77 ± 0.04	0.69 ± 0.05		0.73 ± 0.03	0.63 ± 0.05	
D’amico risk groups									
Low	0.85 ± 0.05	0.76 ± 0.08		0.85 ± 0.05	0.85 ± 0.05		0.81 ± 0.06	0.79 ± 0.07	
Intermediate	0.73 ± 0.08	0.68 ± 0.09	0.211	0.82 ± 0.07	0.73 ± 0.09	0.404	0.78 ± 0.07	0.72 ± 0.09	0.528
Low + Intermediate	0.80 ± 0.04	0.72 ± 0.06		0.83 ± 0.02	0.80 ± 0.05		0.80 ± 0.03	0.76 ± 0.05	
High	0.59 ± 0.06	0.48 ± 0.08	0.001	0.68 ± 0.06	0.51 ± 0.08	<0.001	0.60 ± 0.06	0.47 ± 0.08	<0.001
NCCN risk groups									
Low	0.85 ± 0.05	0.76 ± 0.07		0.85 ± 0.05	0.85 ± 0.05		0.81 ± 0.06	0.79 ± 0.07	
Intermediate	0.70 ± 0.06	0.66 ± 0.06	0.091	0.78 ± 0.06	0.69 ± 0.08	0.159	0.71 ± 0.07	0.63 ± 0.08	0.102
Low + Intermediate	0.77 ± 0.04	0.70 ± 0.05		0.81 ± 0.04	0.77 ± 0.05		0.76 ± 0.04	0.71 ± 0.05	
High	0.59 ± 0.08	0.43 ± 0.09	0.004	0.67 ± 0.07	0.48 ± 0.10	0.002	0.63 ± 0.07	0.46 ± 0.09	0.007

*BFS* biochemical failure-free survival, *DFS* disease-free survival, *HIFU* high-intensity focussed ultrasound, *SE* standard error, *NCCN* National Comprehensive Cancer Network

**Fig. 1:**
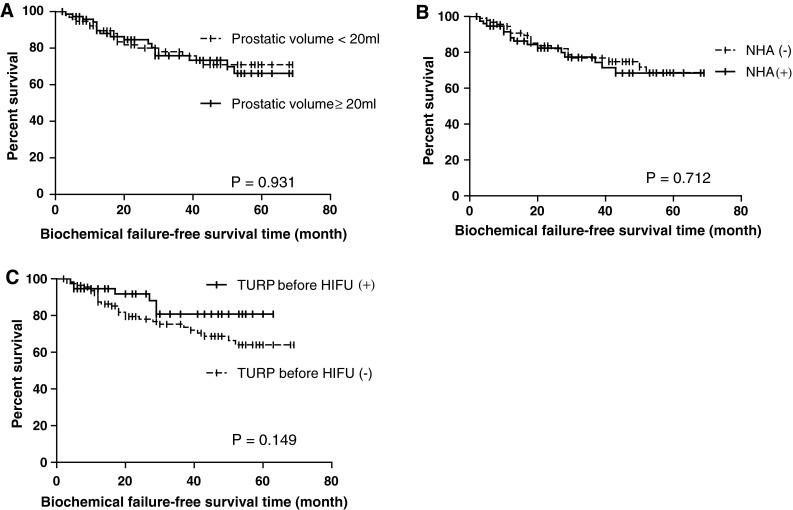
Kaplan–Meier curves of biochemical failure-free survival using Phoenix definition (nadir +2 ng/ml) based on the preoperative variables: **a** prostatic volume immediately before HIFU (cutoff of 20 ml), **b** preoperative NHA, and **c** TURP before HIFU

Additional subgroup analyses were conducted, using Phoenix definition (nadir +2 ng/ml) based on preoperative variables including prostatic volume immediately before HIFU (cutoff of 20 ml), TURP before HIFU and preoperative NHA (Fig. 1). None of them showed significant differences, while there was a tendency that administration of the TURP before HIFU favorably affected cancer control after HIFU, but this tendency did not achieve statistical significance.

### Clinical outcomes

**Fig. 2:**
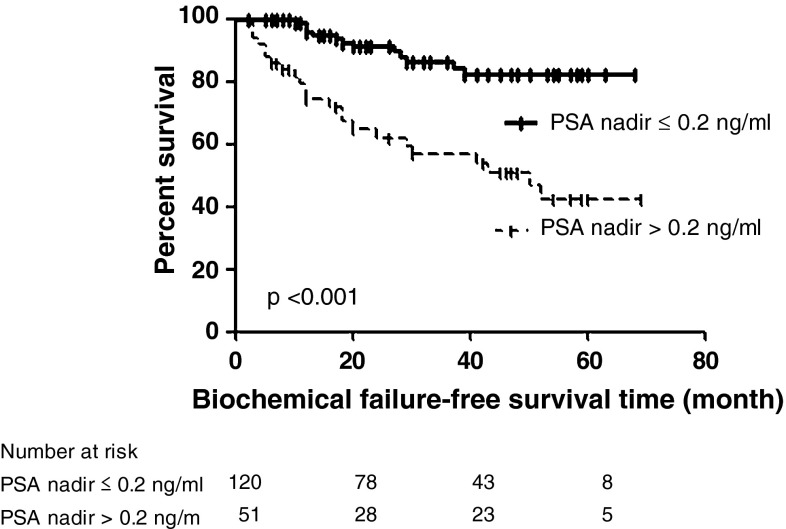
Kaplan–Meier curve of biochemical failure-free survival using Phoenix definition (nadir +2 ng/ml) according to PSA nadir level of 0.2 ng/ml

**Fig. 3:**
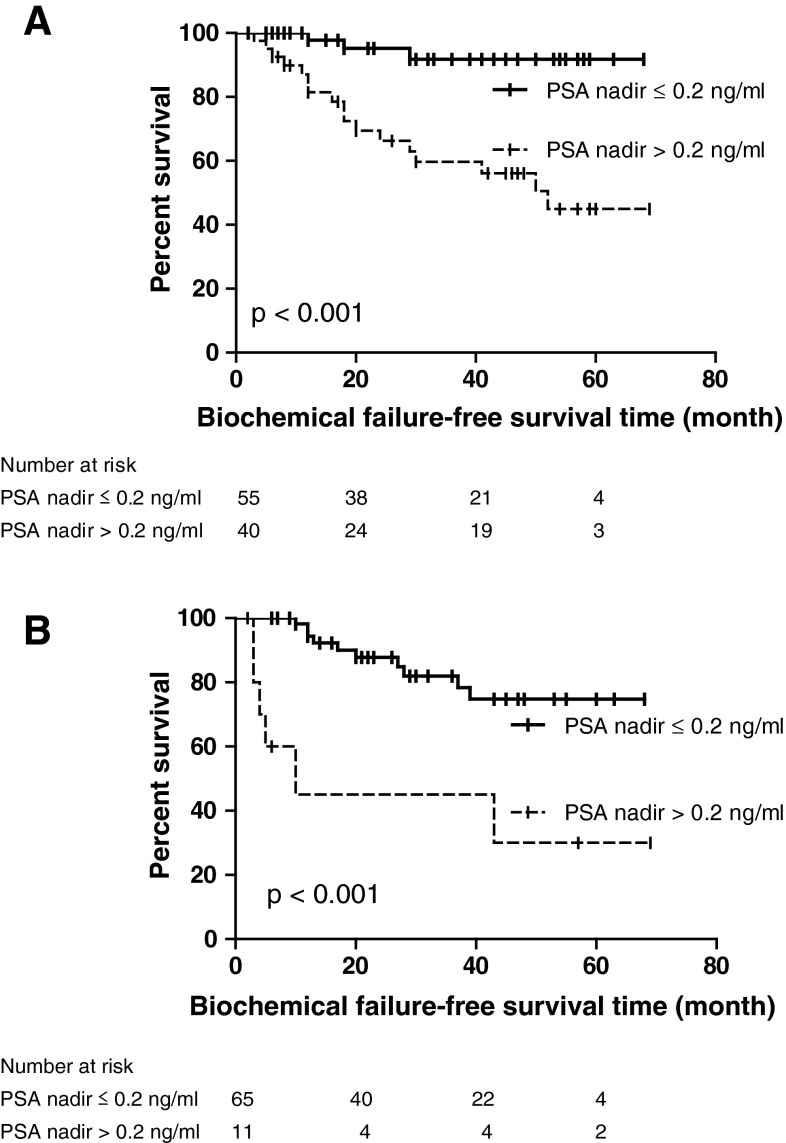
Kaplan–Meier curves of biochemical failure-free survival using Phoenix definition (nadir +2 ng/ml) according to PSA nadir level of 0.2 ng/ml for patients of **a** not having NHA and **b** having NHA

Control biopsy was performed in 103 (60.2 %) patients with 6.5 months of median duration to biopsy with 80.6 % (83/103) of negative biopsy rate. Of 103 patients, 91 (88.3 %) patients achieving PSA threshold level of 1.0 ng/ml were the candidates to undergo follow-up biopsy with 91.2 % (83/91) of negative biopsy rate, whereas all the remaining 11.7 % (12/103) patients not achieving the PSA level of 1.0 ng/ml at 6 months after HIFU had positive follow-up biopsy. Of the all of patients who had negative biopsy, 15.7 % (13/83) patients eventually experienced biochemical relapse for Phoenix definition. A total of 44 (25.7 %) patients (7, 9, and 28 patients for low, intermediate, and high D’amico risk group, respectively) were diagnosed as disease failure with Phoenix definition (nadir +2 ng/ml) in 24 patients and positive follow-up biopsy in 20 patients. Of those, a new HIFU session was offered as salvage therapy in 29.5 % (13/44) patients, hormone deprivation in 47.7 % (21/44) patients, and EBRT in 22.7 % (10/44) patients, respectively (Figs. 2, 3).

### PSA nadir value after HIFU

Median nadir PSA was 0.03 ng/ml (IQR 0.01–0.30) with median time to PSA nadir of 2.5 months (IQR 1.0–3.0). Seventy-six patients (44.4 %) were offered administration of NHA, which would affect the course of PSA value after HIFU. Therefore, we stratified the patients according to the administration of NHA, in which the median nadir PSA level in patients offered NHA was significantly lower than those in patients not offered NHA (0.01 and 0.09 ng/ml, respectively) (*p* = <0.001) and median time to PSA nadir was also significantly shorter in the cohort offered NHA (2.0 months) compared with those not offered NHA (3.0 months) (*p* = <0.001). For the overall cohort, 120 (70.2 %) patients achieved PSA nadir level of ≤0.2 ng/ml, whereas the administration of NHA significantly contributed to achievement of PSA nadir level of ≤0.2 ng/ml (*p* = <0.001).

### Predictive values for biochemical failure

On Cox regression analysis including pre-treatment PSA value, Gleason score, PSA nadir level, clinical T stage, and D’amico risk groups, the predictors for biochemical failure based on the Stuttgart definition after single session were PSA nadir >0.2 ng/ml (HR = 9.472 [95 % CI 4.527–19.820], *p* < 0.001) and D’amico risk groups (HR = 3.132 [95 % CI 1.251–6.389], *p* = 0.033).

## Discussion

In the present study, we investigated the utility of the HIFU device as a primary therapy focused on single-session treatment for the localized prostate cancer. Follow-up monitoring on biochemical relapse for patients treated with HIFU has been conducted applying various definitions which had adaptation for radiation therapy and radical prostatectomy [7, 12–15]. Thus, to obtain integrity of treatment outcomes of HIFU treatment when comparing with previous published studies, we demonstrated the cancer control outcomes applying both biochemical definition including Stuttgart definition, which was derived from the previous studies focused on HIFU, and Phoenix definition, and stratified patients into two risk category including D’amico risk groups and NCCN risk groups.

Pinthus et al. [9] investigated oncological outcomes of single-session HIFU treatment using the Ablatherm^®^ for 402 patients who have not undergone neither NHA nor preoperative TURP, in which they founded that patients with a prostate volume of ≤30 ml had significantly higher BCR-free rate for Stuttgart definition (at 4 years 72 % for a prostate volume ≤30 ml and 56 % for a prostate volume >30 ml, *p* = 0.002), while their median follow-up of 24 months was relatively short and mean prostate volume was 36.7 ml. In the present study with a median follow-up of 43 months, patients offered NHA and preoperative TURP to reduce the prostatic volume and to resect calcification within the prostate were enrolled into the cohort, and prostate volume at the time of HIFU was a mean of 20.1 ml. Although there was neither significant differences for BFSR when stratified patients according to the administration of NHA nor carrying out of preoperative TURP, we could demonstrate that patients with prostate volume of >20 ml at the time of HIFU had statistically equivalent BFSR compared with prostate volume ≤20 ml. When adequately applied to patients before HIFU, these procedures might have additional benefit for biochemical relapse, clinically leading to excellent treatment outcome. In fact, the 5-year BFSR for Stuttgart definition of 72 % for our cohort including low and intermediate risks appears to beyond the 4-year BFSR of 68 % reported by Pinthus et al.

The PSA nadir value has consistently presented as a major predictive factor for treatment success of HIFU [16, 17]. Similarly, we identified the PSA nadir value as the independent predictor for biochemical failure of Stuttgart definition after single-session HIFU using the Sonablate^®^. However, concerning that the administration of NHA would affect the nature course of PSA after HIFU has made those previous studies disinclined to include patients who underwent NHA. Nevertheless, as described previously, we believe that offering NHA is practically essential to reduce prostate volume leading to durable cancer control.

The present study also represented that median time to PSA nadir level after single-session HIFU was 2.5 months allowing an early feedback on treatment efficacy compared with that after EBRT which is usually achieved after 18 months [18, 19]. Additionally, patients who presented a local relapse could be followed by a later using salvage radiation therapy, which might explain the good cancer control after single-session HIFU achieved in 99.4 % of the metastasis-free survival rate and 100 % of CSSR at 5 years.

Rebillard et al. [20] reported negative biopsies rate after treatment with the Ablathern^®^ device reaches 90 % in patients with low- and intermediate-risk disease who underwent routine post-HIFU prostate biopsy. Even though executing rate of follow-up biopsy (60.2 %, 103/171) in our cohort might be relatively low, we could eventually identified 88.3 % (91/103) of patients who presented the threshold PSA level of <1.0 ng/ml at the point of biopsy and revealed 91.2 % (83/91) of negative biopsy rate in those patients, which could have a potential closer to the true treatment outcome than any tracking of biochemical measurements. Crouzet et al. [3] mentioned that the additional treatment survival rate is more accurate to present the real clinical outcomes after HIFU, and the combination of the Phoenix criteria and additional treatment survival, defined as DFSR, represents the real HIFU outcomes, estimating for 72 % in low-. 56 % in intermediate-, and 47 % in high-risk patients. In our findings, the biochemical relapse for low- and intermediate-risk patients seems to be comparable to those previous reports but inferior for high-risk patients while comparing favorably with any risk groups for the DFSR, defined as biochemical relapse for Phoenix definition or positive follow-up biopsy in the present study, including high-risk patients. Interestingly, those previous studies could consider patients experienced new HIFU session as both biochemical- and disease-free. We believe that repeated HIFU treatment might offer the additional benefit in patients with high-risk disease, and the administration of hormone deprivation or EBRT as salvage therapy for the high-risk disease would probably result in the similar DFSR comparing our single-session HIFU treatment outcomes. Moreover, we also believe that HIFU may represent the first step of a multimodal treatment approach in patients with high-risk disease.

## Conclusion

We firstly assessed the treatment efficacy of single-session HIFU treatment using the Sonablate^®^ for patients with localized prostate cancer. Well-formed application of NHA and preoperative TURP for patients with larger volume and calcification of prostate would conduce toward the treatment success where primary HIFU therapy could advance to a new stage the first step among the multimodal treatment including later radiation, radical prostatectomy in patients even with high-risk disease. To verify these findings, further well-designed prospective study is needed.
